# Data about knowledge and tendency towards organic foods use in Tehran

**DOI:** 10.1016/j.dib.2017.12.033

**Published:** 2017-12-18

**Authors:** Fariba Razeghi, Ehsan Haghi, Masoud Yunesian

**Affiliations:** a Department of Environmental Health Engineering, School of Public Health, Tehran, University of Medical Sciences, Tehran, Iran; b Department of Environmental Health Engineering, School of Public Health, Tehran University of Medical Sciences, Tehran, Iran

## Abstract

Improper use of chemical fertilizer and pesticide poses not only threats to the environmental safety but also major public health issues globally. The adverse effects of chemical fertilizers and pesticides forced agricultural scientist to look for safer methods such as organic farming. This study was aimed at assessing the knowledge and tendency towards organic foods use among people of living in a megacity, Tehran. Data was collected from “fall exhibition” and “health food exhibition” participants using pretested questionnaire. Data were entered, cleaned and analyzed by SPSS version 17. *T*-test, ANOVA and Regression analysis were carried out and the association was considered significant at *p*-value less than 0.05. A total of 400 respondents participated in the study, making a response rate of 100%. There were reverse relation between knowledge and accessibility and positive relation between trust, marriage and gender and no relation with price. Building trust in consumer, and allocation of a special label, known logos and ways to track most of the products sold as organic foods seems necessary for increasing consumption.

**Specifications Table**

**Table:** 

Subject area	*Food safety, Agriculture*
More specific subject area	***Data analysis about knowledge and tendency towards organic foods use in Tehran***
Type of data	*Table, text file*
How data was acquired	*survey*
Data format	*Raw data statistically analyzed*
Experimental factors	*Does not apply*
Experimental features	*The questionnaire contained three sections that obtained data concerning the participants’ demographic characteristics, knowledge about organic foods, and tendency towards organic foods use. Data were entered, cleaned and analyzed by SPSS version 17. T-test, ANOVA and Regression analysis were carried out and the association was considered significant at p-value less than 0.05. A total of 400 respondents participated in the study, making a response rate of 100%.*
Data source location	*Tehran, Iran*
Data accessibility	*Analyzed data is available in this article*

**Value of the data**

• The findings can serve as input to the multimedia to inform the public about the organic foods and their benefits and prevention of counterfeit products.
• The better and true understanding of the consumers’ food behavior can improve the markets for organic foods products.
• Encourage people and consumer to buy more organic food products.

## 1 Data

The agriculture sector uses chemical fertilizers to increase agricultural production to enable feeding the growing food demands of populations [1]. In Iran, chemical fertilizer utilization increased from 2.4 t in 2001 to 3.3 t in 2011. That means more than 27,000 t chemical fertilizers have been used in agriculture sector annually [2]. In 2010, Iran was ranked 93rd in the world due to improper use of chemical fertilizers, hormones, pesticides and chemical residuals in agricultural products [3].

This study aims to assess the knowledge and tendency towards organic foods use among 400 people who randomly selected from two exhibitions (fall and healthy food exhibitions) held in Tehran, Iran. The respondents had forty-six percent (46%) of the knowledge about organic food and were able to correctly describe it, whilst 45% did not. among the healthy foods exhibition participants, these figures were 63% and 24% respectively (*p*=.001). Table 1 shows Description figures about tendency towards organic foods use, there was no statistically significant difference between the two groups in relation to their tendency towards organic foods use. Table 2 shows ANOVA between independents variables and tendency towards organic food and Table 3 indicates regression coefficients model for independent variables. There were reverse relation between knowledge and accessibility and positive relation between trust and no relation with price.

**Table 1:** Description figures about tendency towards organic foods use.

Variables	Degree of freedom	Average of squares	*F*	*P* values
Knowledge	1	746.42	44.08	<0.001
Trust	1	3279.67	310.34	<0.001
Accessibility	1	76.17	4.09	0.044
Price	1	47.95	2.56	0.11

**Table 2:** ANOVA between independents variables and tendency towards organic food.

Tendency towards organic foods	**Healthy food exhibition**		**Fall exhibition**		*P* values
	Frequency	Percent	Frequency	Percent	
Weak tendency	0	0	1	5	<0.001
Middle tendency	154	77	168	84	
Strong tendency	46	23	31	15.5	
Total	200	100	400	100

**Table 3:** Regression coefficients model for independent variables.

		Coefficients	*t*	*P* values
	*β*	Standard errors		
Constant	12.11	.229	98.52	<0.001
Knowledge	−.87	.132	−6.63	<0.001
Constant	10.54	.206	51.2	<0.001
Trust	1.42	.081	17.61	<0.001
Constant	12.009	.436	27.55	0.001
Accessibility	−.19	.094	−2.02	.044

## 2 Experimental design, materials and methods

This is a descriptive-analytical study that utilized data collected through questionnaires adapted from previous studies [3], [4]. The questionnaire contained three sections that obtained data concerning the participants’ demographic characteristics, knowledge about organic foods, and tendency towards organic foods use. Food safety specialist reviewed the questionnaire and modifications were made accordingly before its administration. Then, it was further pretested 50 randomly selected individuals. The analysis of the responses provided Cronbach's alpha test result of 70%, indicating good reliability of the questionnaire.

The study was conducted on a randomly selected individuals from two exhibitions held in Tehran. One of the exhibitions was about “fall exhibition” that mainly presented school articles and the other was on “healthy food” that presented organic food products. We computed the sample size by the formula for simple random sampling technique using the proportion (*p*=0.5) that provides the maximum sample size (because we did not get the proportion that had knowledge about healthy foods from studies on exhibitions in a similar context), at 95% level of significance, and margin of error (d) of 0.05. The sample size was calculated at 384, 10% was added, as a contingency for non-response, and the final sample was settled at 400. The data was collected from 200 individuals each from the two exhibitions in the morning and in the evening sessions. Different analytical techniques including *T*-test, ANOVA and regression analysis were carried out using the statistical packages for socials sciences (SPSS) version 17.
